# Cordycepin induces apoptosis by caveolin-1-mediated JNK regulation of Foxo3a in human lung adenocarcinoma

**DOI:** 10.18632/oncotarget.14661

**Published:** 2017-01-14

**Authors:** Jong Cheon Joo, Jung Hoo Hwang, Eunbi Jo, Young-Rang Kim, Dae Joon Kim, Kyung-Bok Lee, Soo Jung Park, Ik-Soon Jang

**Affiliations:** ^1^ Department of Sasang Constitutional Medicine, Wonkwang University, Iksan, 54538, Republic of Korea; ^2^ College of Medicine, Chung-Ang University, Seoul 156-756, Republic of Korea; ^3^ Division of Bioconvergence Analysis, Korea Basic Science Institute, Daejeon 305-333, Republic of Korea; ^4^ Department of Biomedical Sciences, School of Medicine, University of Texas Rio Grande Valley, Edinburg, TX 78539, USA; ^5^ Department of Sasang Constitutional Medicine, Woosuk University, Wanju, Jeonbuk, 55338, Republic of Korea

**Keywords:** cordycepin, CAV1, JNK, Foxo3a, apoptosis

## Abstract

Forkhead transcription factor (Foxo3a) is a downstream effector of JNK-induced tumor suppression. However, it is not clear whether the caveolin-1 (CAV1)-mediated JNK/Foxo3a pathway is involved in cancer cell apoptosis. We found that cordycepin upregulates CAV1 expression, which was accompanied by JNK phosphorylation (p-JNK) and subsequent Foxo3a translocation into the nucleus, resulting in the upregulation of Bax protein expression. Furthermore, we found that CAV1 overexpression upregulated p-JNK, whereas CAV1 siRNA downregulated p-JNK. Additionally, SP600125, a specific JNK inhibitor, significantly increased Foxo3a phosphorylation, which downregulated Foxo3a translocation into the nucleus, indicating that CAV1 mediates JNK regulation of Foxo3a. Foxo3a siRNA downregulated Bax protein and attenuated A549 apoptosis, indicating that the CAV1-mediated JNK/Foxo3a pathway induces the apoptosis of A549 lung cancer cells. Cordycepin significantly decreased tumor volume in nude mice. Taken together, these results indicate that cordycepin promotes CAV1 upregulation to enhance JNK/Foxo3a signaling pathway activation, inducing apoptosis in lung cancer cells, and support its potential as a therapeutic agent for lung cancer.

## INTRODUCTION

Cordycepin is an adenosine analog that is readily phosphorylated to its mono-, di-, and triphosphates intracellularly. Cordycepin activity has been well described *in vitro* using purified RNA polymerases and poly(A) polymerases from a number of organisms, including yeasts and mammals [1]. Cordycepin significantly inhibits cell growth by inducing apoptosis through a signaling cascade involving the caspase pathway [2], and selectively induces apoptosis in MA-10 mouse Leydig tumor cells via p38 MAPK signaling [3]. Three MAPK pathways have been identified to date: the extracellular signal-regulated protein kinase pathway, the JNK pathway, and the p38 MAPK signaling pathway [4]. JNK modulates Foxo3a to promote mitochondrial death [5]. Cordycepin mediates apoptosis by increasing SAPK/JNK and p38 MAPK activities and by upregulating the expression of Bcl-2 pro-apoptotic proteins. Studies also have shown that cordycepin has significant anti-tumor effects such as inhibition of cell growth and metastasis [6, 7] and it interferes with various cell-signaling pathways [8, 9]. Cordycepin is one of the 18 new anti-cancer drugs currently under investigation and in development by the National Cancer Institute in the USA [10]. However, the molecular mechanism by which cordycepin inhibits tumor cell growth and induces apoptosis remains unclear.

The role of caveolin-1 (CAV1) in cancer is highly controversial. CAV1 expression is reduced in a variety of human tumors [11, 12] and CAV1 re-expression is often sufficient to attenuate functions associated with the transformed phenotype in cancer cells [11–13]. Moreover, CAV1 knockout mice exhibit increased angiogenesis and predisposition to tumor proliferation, underscoring a role for CAV1 in tumor suppression [14]. In contrast to these observations, the presence of CAV1 reportedly facilitates more aggressive traits in several cancer cell lines, and is related with metastasis, drug resistance, and poor prognosis [15, 16]. However, since it has not been proven that cordycepin regulates the CAV1-dependent pro-apoptotic pathway, further study is needed.

In this study, we analyzed the effects of cordycepin on lung cancer cell apoptosis and studied the relationship between CAV1 and JNK. We attempted to identify the pathway by which cordycepin promotes CAV1-mediated JNK/Foxo3a signaling, thereby inducing apoptosis in human lung-cancer cells. The data presented herein clearly show that cordycepin is involved in the JNK/Foxo3a signaling pathway by stimulating CAV1 signaling, and that the consequent activation of Bax/caspase-3-mediated pathway causes cancer cell death.

## RESULTS

### Cordycepin inhibits lung cancer cell growth

**Figure 1:**
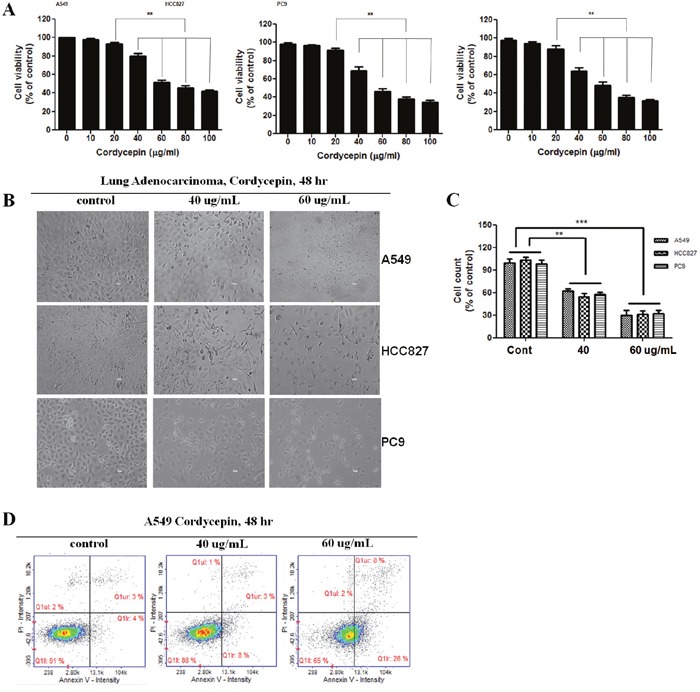
Cordycepin induces apoptosis in lung cancer cells **A**. Inhibition of the growth of lung cancer cells by cordycepin. A549 cancer cells were exposed to cordycepin at 0, 20, 40, 60, 80, or 100 μg/mL for 48 h. Data are presented as the mean ± standard deviation from triplicate experiments. *p < 0.05 and **p < 0.01 vs. untreated control. **B**. Micrographs of A549 cells treated with 40 or 60 μg/mL of cordycepin for 48 h. Magnification 400×. **C**. Cell counts (% of control) after treatment with cordycepin at 0, 40, or 60 μg/mL for 48 h. **D**. Apoptosis analysis of A549 cells exposed to cordycepin.

To investigate the effects of cordycepin on lung cancer cell proliferation, A549, HCC827, and PC9 cells were treated directly with 0, 10, 20, 40, 60, 80, or 100 μg/mL for 24 h and 48 h. As shown in Figure 1A, cordycepin inhibited the cell growth during the 48-h incubation in a dose-dependent manner. At 60 μg/mL, cordycepin inhibited approximately half of all three lung cancer cell populations. Thus, the half-maximal inhibitory concentration (IC_50_) was determined as 60 μg/mL cordycepin (Figure 1A). To observe the cell death of cordycepin-treated cancer cells, the morphologies of lung cancer cells were compared to those of untreated control cells by using light microscopy. The morphology of A549, HCC827, and PC9 cells changed drastically after 60 μg/mL cordycepin treatment for 48 h (Figure 1B, 1C). Multiple cells began to detach from the surface of the culture plate and appeared buoyant. Moreover, the cells appeared to be shrunken, resulting in reduced cell volume. These morphological changes preceded apoptosis. On the other hand, 40 μg/mL cordycepin induced less drastic changes at 48 h.

### Cordycepin induces apoptosis in lung adenocarcinomas

The Cordycepin apoptotic effect on A549 lung cancer cells was analyzed with Annexin V- and PI-stained cells using flow cytometry after 48-h treatment with 40 or 60 μg/mL cordycepin. The assay was done to evaluate how cancer cell death was induced by cordycepin. The relative proportion of non-viable cells was quantitatively measured as cells at the early stage of apoptosis (Annexin V-stained, non-disrupted cells) or as cells entering the late stage of apoptosis (disrupted or lysed cells). In 40 μg/mL cordycepin-treated cells, no drastic change in the Annexin V-stained viable fraction was observed (91% to 88%) (Figure 1D). However, the cells treated with 60 μg/mL cordycepin were markedly shifted from the normal state to the early apoptotic stage (4% to 26%), whereas the viable fraction was reduced from 91% to 65%. Thus, 60 μg/mL cordycepin treatment induces the apoptotic process in lung cancer cells.

### Cordycepin alters gene expression

**Figure 2:**
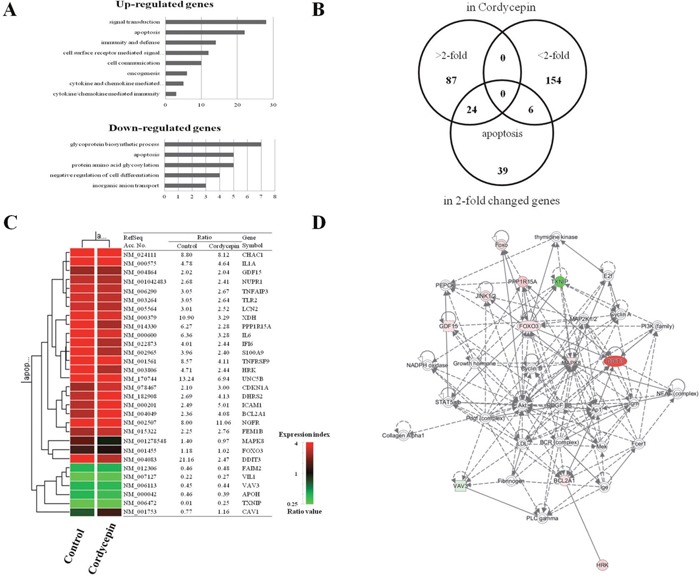
Gene expression analysis and signal network of apoptotic genes **A**. Results of microarray analysis of gene expression in response to 60 μg/mL of cordycepin for 48 h. Genes showing twofold upregulation or downregulation identified for Gene Ontology analysis **B**. Venn diagram of gene expression in response to cordycepin genes that were altered more than twofold and apoptosis-related genes. **C**. Apoptosis-related genes lists that were altered more than twofold in response to cordycepin. **D**. Signal network of the apoptotic genes generated by using a Qiagen IPA.

To identify the genes potentially involved in the anti-cancer activity of cordycepin, we conducted microarray analysis of A549 cancer cells after treatment with 60 μg/mL cordycepin. Among the 63,242 unique genes (using the Agilent Human GE 8×60K Microarray) tested, 30,858 genes were expressed in the cordycepin-treated cells. Among these 30,858 genes, 2,561 and 1,942 genes were up- and downregulated, respectively, by 48-h treatment with cordycepin. Genes that were significantly up- or downregulated by more than 2-fold were subjected to GO enrichment analysis using the Database for Annotation, Visualization, and Integrated Discovery (DAVID) tools (http://david.abcc.ncifcrf.gov/). It was showed more than a 2-fold changed genes through GO analysis (Supplementary Table 1). The upregulated genes were mainly involved in signal transduction, immunity and defense, cell surface receptor-mediated signaling, cell communication, apoptosis, ligand-mediated signaling, cell adhesion-mediated signaling, natural killer cell-mediated immunity, and B cell- and antibody-mediated immunity (Figure 2A). The downregulated genes were related to transport, ion transport, cell adhesion, apoptosis, homeostasis, and phosphate metabolism. To identify genes potentially involved in apoptosis among cordycepin-induced genes, we used the GeneCards database (http://www.genecards.org/) (Figure 2B, 2C). There were quantitative alterations in gene expression in cordycepin-treated lung cancer cells as compared to control cells. The signal network of apoptotic genes regulated in response to cordycepin is shown in Figure 2D. Among these, CAV1 and JNK (MAPK8) were identified as central hubs of the apoptosis-related interactome network in the cordycepin-treated lung cancer cells.

### Cordycepin increases CAV1 and p-JNK expression and prevents phosphorylation of Foxo3a, inducing Foxo3a nuclear translocation

**Figure 3:**
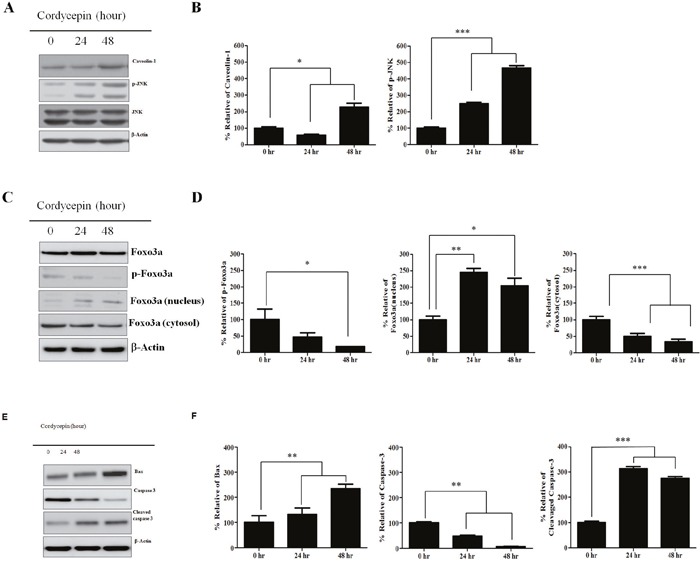
Expression of CAV1, p-JNK, JNK, Foxo3a, nuclear Foxo3a proteins in response to cordycepin **A**. Western blots showing expression of caveolin-1, p-JNK, JNK, and **B**. relative band intensities of CAV1, p-JNK, JNK **C**. Total Foxo3a, p-Foxo3a, nuclear and cytosolic Foxo3a, and **D**. relative band intensities of p-Foxo3a, nuclear, and cytosolic Foxo3a **E**. Bax, caspase-3, and cleaved caspase-3 at 0, 24, and 48 h of cordycepin treatment of A549 cells, and **F**. relative band intensities of Bax, caspase-3, and cleaved caspase-3. Results are shown as mean ± S.E.M. Data were analyzed using Student's *t*-test. *p <0.05, **p < 0.01 and ***p < 0.001 vs. control.

CAV1, p-JNK (active JNK), and total JNK expression at 24 and 48 h after cordycepin treatment was analyzed by western blot (Figure 3A, 3B). Total JNK protein was not changed each different time points when compared to the control. However, CAV1 and p-JNK protein expression increased over time after cordycepin treatment. Because Foxo3a has been shown as a primary substrate of JNK in tumor growth inhibition, we assessed whether Foxo3a was changed at 24 h and 48 h after cordycepin treatment. We quantified both total Foxo3a and p-Foxo3a protein expression (Figure 3C, 3D). Total Foxo3a was not significantly changed at the indicated time points. However, p-Foxo3a markedly decreased over 48 h by cordycepin treatment. Foxo3a dephosphorylation has been reported to accelerate its nuclear translocation.

We assessed whether cordycepin-induced Foxo3a dephosphorylation would facilitate Foxo3a translocation into the nucleus. We extracted the nuclear and cytosolic proteins from A549 cells and quantified Foxo3a expression separately in both fractions by western blot analysis (Figure 3C, 3D). We found that nuclear Foxo3a increased in a time-dependent manner. In contrast, cytosolic Foxo3a was significantly decreased at 24 h and 48 h. To determine whether Bax, as a downstream target of Foxo3a was upregulated by Foxo3a nuclear translocation, we quantified Bax protein expression using protein immunoblot analysis (Figure 3E, 3F). We found that Bax protein was upregulated at 24 h and 48 h after cordycepin treatment. Cleaved caspase-3 is a key executor of the apoptosis cascade reaction.

Western blot analysis indicated that cleaved caspase-3 protein was upregulated at 24 h and 48 h (Figure 3E, 3F).

### CAV1 phosphorylates JNK and regulates Foxo3a nuclear translocation

**Figure 4:**
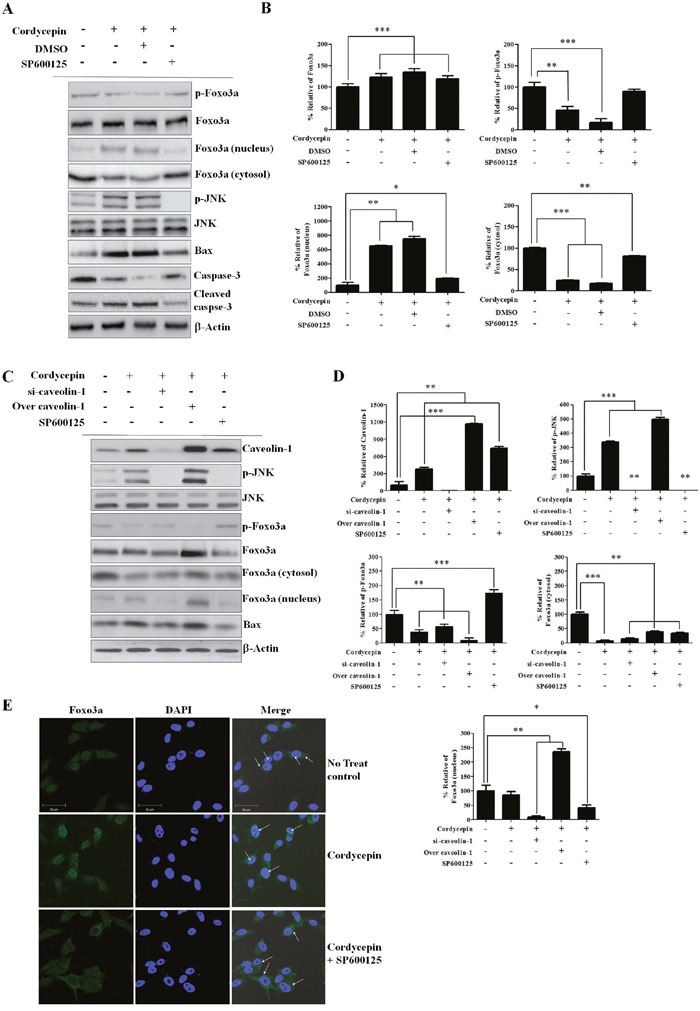
CAV1 phosphorylates JNK and regulates nuclear Fox3a translocation **A**. Representative western blots of A549 cells No treated control and 60 μg/mL of cordycepin alone or in combination with DMSO or the JNK inhibitor 10 mM of SP600125 **B**. Relative band intensities of Foxo3a, p-Foxo3a, cytosolic Foxo3a, and nuclear Foxo3a. **C**. Western blots of CAV1, p-JNK, JNK, Foxo3a, p-Foxo3a, cytosolic Foxo3a, nuclear Foxo3a. A549 cells were incubated with si-CAV1 or negative control siRNA for 48 h, transfected with a CAV1-overexpression construct for 48 h, or treated with SP600125 for 48 h. **D**. Relative band intensities of p-JNK, p-Foxo3a, cytosolic Foxo3a, nuclear Foxo3a, CAV1, Bax. **E**. A549 cells were grown on glass coverslips and treated with cordycepin or cordycepin with 10 mM of SP600125 for 48 h. Samples were analyzed by indirect immunofluorescence with confocal microscopy. Arrows indicate the nuclei. The bar indicates 50 μm.

To confirm whether JNK is involved in Foxo3a regulation after cordycepin treatment, we used SP600125, a highly specific JNK inhibitor. In A459 cells treated with 10 mM of SP600125, there was an increase in the level of p-JNK 48 h after cordycepin treatment as compared to the controls (Figure 4A, 4B). Next, we investigated whether SP600125 affected Foxo3a expression and phosphorylation. SP600125 treatment significantly increased the expression of p-Foxo3a at 48 h after cordycepin treatment (Figure 4A, 4B). However, total Foxo3a was not changed at 48 h by SP600125 treatment. Because SP600125 upregulated p-Foxo3a after cordycepin treatment, we further investigated whether JNK was involved in the regulation of Foxo3a translocation from the cytoplasm to the nucleus. As expected, nuclear Foxo3a was upregulated while the cytoplasmic fraction was downregulated at 48 h after cordycepin treatment (Figure 4A, 4B). However, SP600125 treatment suppressed Foxo3a nuclear translocation as compared to the DMSO-treated control (Figure 4A, 4B).

We assessed whether CAV1 was involved in the positive regulation of p-JNK after cordycepin treatment by siRNA-mediated silencing of CAV1. Cordycepin increased the expression of CAV1 and p-JNK, whereas siRNA-mediated inhibition of CAV1 effectively lowered the CAV1 protein level and abrogated p-JNK expression indicating that CAV1 is involved in the upregulation of JNK phosphorylation (Figure 4B). Additionally, we determined whether CAV1 affects the expression of (nuclear and cytoplasmic) Foxo3a (Figure 4C, 4D). Overexpression of CAV1 through CAV1-GFP lentivirus decreased the expression of total p-Foxo3a and the cytosolic Foxo3a fraction, whereas it increased nuclear Foxo3a protein. In contrast, siRNA-mediated inhibition of CAV1 increased the expression of p-Foxo3a and cytosolic Foxo3a, whereas it decreased nuclear Foxo3a protein. These results indicated that CAV1 upregulates Fox3a nuclear translocation. This translocation was also observed in immunofluorescence assays. Under non-stimulated condition, Foxo3a is present in the cytoplasm and nucleus. As shown in Figure 4E, green fluorescence-tagged Foxo3a was indeed localized in the cytoplasm and near the nucleus of untreated A549 cells. When the cells were stimulated with cordycepin (60 μg/mL, 48 h), Foxo3a was translocated to the nucleus. SP600125 treatment significantly decreased the expression of nuclear Foxo3a at 48 h after cordycepin treatment as compared to the cordycepin-alone control. This result suggested that cordycepin promotes Foxo3a signaling to induce apoptosis via JNK activation in lung cancer cells.

### Cordycepin upregulates JNK phosphorylation through CAV1-mediated DUSP5 inhibition

**Figure 5:**
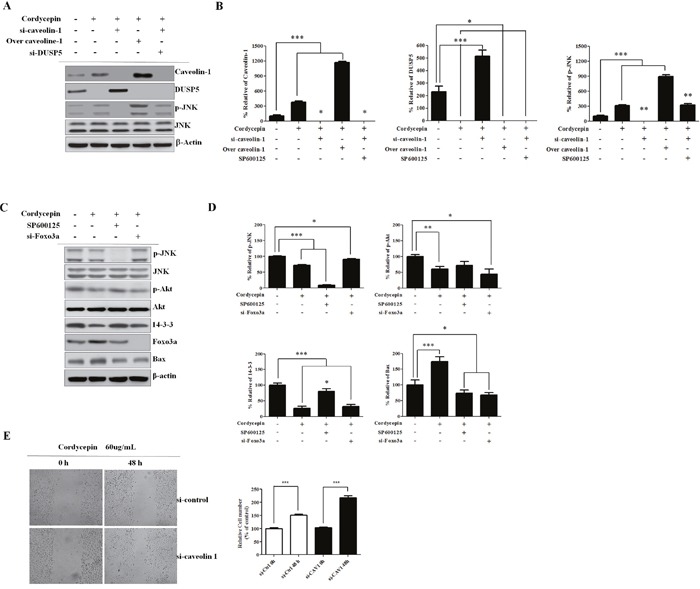
Knockdown of CAV1 leads to decreased p-JNK and Foxo3a is regulated by Akt and 14-3-3 **A**. Western blots of CAV1, DUSP5, p-JNK, and JNK. A549 cells were incubated with siRNA (si-CAV1) for 48 h, transfected with a CAV1-overexpression construct, or treated with siRNA against DUSP5 (si-DUSP5). CAV1-silenced A549 cells treated with cordycepin showed increased DUSP5 and decreased p-JNK, and siRNA inhibition of DUSP5 in these cells increased p-JNK as compared to control cells. **B**. Relative band intensities of CAV1, DUSP5, p-JNK/JNK ratio. **C**. Representative western blots of JNK, pAkt, Akt, 14-3-3, and Foxo3a in A549 cells treated with cordycepin, SP600125, or siRNA targeting Foxo3a (si-Foxo3a). **D**. Relative band intensities of p-JNK, p-Akt, 14-3-3, Bax. **E**. Migration ability of A549 cells as examined by wound-healing assay. Migrating cells were photographed under an inverted fluorescence microscope (magnifications: 100×). Quantification of the numbers of migrating are presented as the mean ± standard deviation of three independent experiments performed in triplicate. *p <0.05, **p < 0.01, ***p < 0.001 vs. negative control (0 μg/mL).

To elucidate how CAV1 upregulation enhances JNK activation in more detail, we conducted western blot analysis in A549 cells. In doing so, we clarified alterations in the JNK/Foxo3a signaling pathway (Figure 5A, 5B). siRNA inhibition of CAV1 in A549 cells treated with cordycepin resulted in increased DUSP5 and decreased p-JNK levels, while silencing of DUSP5 increased p-JNK. DUSP5 is a nuclear protein that negatively regulates JNK. These results indicated that cordycepin upregulates JNK phosphorylation through CAV1-mediated DUSP5 inhibition.

### The cordycepin-induced increase in Foxo3a upregulates Bax and Akt and 14-3-3 activities

There is crosstalk between the pro-apoptotic JNK and the pro-survival Akt pathways at various levels. To further clarify whether the regulation of Foxo3a by JNK after cordycepin treatment is associated to Akt, we assessed p-Akt and Akt protein expression after cordycepin treatment by western blotting. Additionally, we silenced Foxo3a using specific siRNA, and we blocked JNK activation with SP600125. We found that both 14-3-3 and p-Akt were decreased, whereas total Akt was not changed at 48 h in the SP600125-treated A549 cells as compared to the non-treated control (Figure 5C, 5D). We investigated whether Bax is regulated by Foxo3a after cordycepin treatment. siRNA-mediated inhibition of Foxo3a slightly decreased Bax expression, indicating the upregulation of Bax by nuclear Foxo3a.

### Coexpression of CAV1 and p-JNK correlates with decreased survival in lung cancer cells

**Figure 6:**
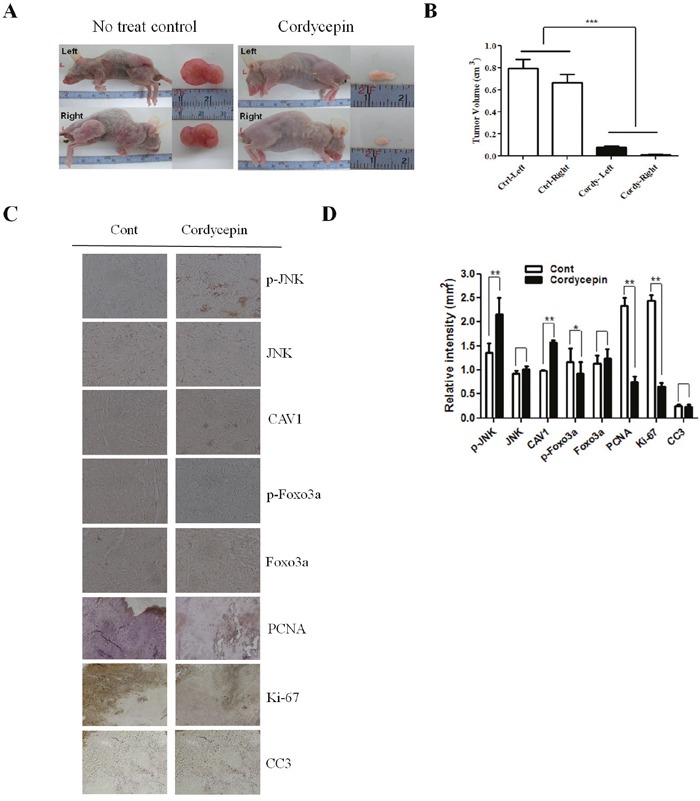
Effect of cordycepin on expression of CAV1, p-JNK, JNK, p-Foxo3a and Foxo3a in lung tumor tissue **A**. Nude mice were injected with A549 cells and then treated with PBS (n = 6) or 20 mg/kg cordycepin (n = 6) once daily for 20 days. Dissected tumors are shown in the right panel. Tumor volumes of control and cordycepin-treated mice. **B**. Bar graph showing that the tumor volume in the cordycepin-treated group was lower than that in the non-treated control group. **C**. Tumor tissues were collected for immunohistochemistry of CAV1, p-JNK, JNK, p-Foxo3a, and Foxo3a as described in the Materials and Methods. Representative photomicrographs of five independent tumor samples are shown. **D**. Quantification of CAV1, p-JNK, JNK, p-Foxo3a, and Foxo3a. Data are presented as the mean ± standard deviation of three independent experiments performed in triplicate. *p < 0.05 and ** *p* < 0.01 vs. the control.

Cordycepin induced apoptosis via CAV1-mediated JNK activation in A549 cancer cells. To evaluate the potential biological relevance of the regulation of CAV1, we assessed the effect on the directed migration of tumor cells. Migration of A549 cells was measured using a wound-healing assay. As shown in Figure 5E, A549 migration was significantly suppressed by cordycepin (*p* < 0.05), whereas CAV1 silencing significantly recovered the migration of A549 cells treated with cordycepin. As cordycepin upregulated CAV1 expression and JNK phosphorylation, and inhibited phosphorylation of Foxo3a *in vitro*, we next examined CAV1, the phosphorylation status of JNK, total JNK, p-Foxo3a, and total Foxo3a protein in tumor tissues by immunohistochemistry. Treatment of mice with cordycepin significantly suppressed tumor growth (Figure 6A, 6B) and upregulated CAV1 and JNK phosphorylation, whereas it inhibited the phosphorylation of Foxo3a and only slightly increased the total Foxo3a protein level as compared to untreated control (Figure 6C, 6D). These data suggest that cordycepin can cause growth arrest in tumor cells *in vivo* by inducing the expression of CAV1 and p-JNK, resulting in downregulation of Foxo3a phosphorylation.

## DISCUSSION

Cordycepin has been reported to have various biological activities, including the inhibition of inflammation, platelet aggregation, mRNA polyadenylation [17, 18], and reinforcement of the immune system [19]. In addition, it has remarkable anti-tumor effects, such as inhibition of cell proliferation [20, 21], induction of apoptosis, and inhibition of cell migration and invasiveness [22]. These antitumor effects have been shown in several organs, such as, pulmpnary carcinoma, bladder, prostate, liver, and gastrointestinal cancer, and mainly involve the induction of apoptosis via the targeting of biologically important molecules and pathways [8, 9]. However, the roles of CAV1 and JNK in the induction of apoptosis remain unclear. This study showed that treatment with 60 μg/mL cordycepin reduced cell viability and strongly inhibited the growth of A549 lung cancer cells (Figure 1A). Moreover, 60 μg/mL cordycepin had a clear effect on the morphology of A549 cells, and the effect was stronger than that of 40 μg/mL cordycepin (Figure 1B, 1C). The results of Annexin V/PI staining using FACS demonstrated that cordycepin could induce pro-apoptosis. Cordycepin at 60 μg/mL, but not 40 μg/mL, induced the transformation of cells from the normal state (untreated group: 91.0% normal, 4.0% early apoptotic, and 3% late apoptotic) to the apoptotic state (65.0% normal, 26.0% early apoptotic, and 8.0% late apoptotic) (Figure 1D). These results suggest that cordycepin exhibits anti-lung cancer activity by promoting pro-apoptosis.

To analyze cordycepin-related gene expression in lung cancer cells, we used a cDNA microarray approach. Clustering of the microarray data identified groups of genes that were differentially regulated upon treatment of the A549 cells with 60 μg/mL of cordycepin. The GO categories of genes whose expression changed by at least two-fold are shown in Figure 2A. Among these, 26 genes whose expression increased and 7 with decreased expression were related to apoptosis (Figure 2B, 2C). To explore the major cordycepin-regulated proteins identified using GO analysis, we used IPA to query 33 proteins that were up- or downregulated by cordycepin, yielding a distinct interconnected network of 27 proteins (Figure 2D). Among these, CAV1/JNK (MAPK8) were the center of the apoptosis-related protein network. After RT-PCR result, CAV1 with cordycepin treatment has slightly increased in both concentration dependent and time dependent manners. These are the same results that shown on Figure 2 with microarray. Therefore, the increase of CAV1 seems that there was a correlation with cell viability that shown in Figure 1A. From the IPA network analysis of microarray results, the increase of CAV1 has a crucial role to induce apoptois of lung adenocarcinoma with cordycepin-treatment. The network analysis of Figure 2D has shown the important relationship between JNK and Foxo3, and the Supplementary Figure 2 data analysis shown that there was very important relationship between CAV1 and JNK (Supplementary Figure 2).

In present study, we showed that the CAV1/JNK/Foxo3a pathway was involved in A549 cell apoptosis after cordycepin treatment. First, we found that CAV1-mediated p-JNK upregulation and the reduction of p-Foxo3a preceded A549 lung cancer cell apoptosis, suggesting that JNK phosphorylation and Foxo3a dephosphorylation were involved in the mediation of cordycepin-induced A549 cell apoptosis. Second, p-Foxo3a downregulation induced Foxo3a nuclear translocation, which upregulated the levels of pro-apoptosis proteins such as Bax and cleaved caspase-3. Third, cordycepin upregulated JNK phosphorylation through CAV1-mediated DUSP5 inhibition. Fourth, treatment with the JNK inhibitor SP600125 significantly increased p-Foxo3a, leading to a decrease in Foxo3a nuclear translocation and in the protein levels of Bax and cleaved caspase-3. Finally, siRNA inhibition of Foxo3a attenuated Bax after cordycepin treatment.

Previous studies of the expression and function of CAV1 in cancer have shown controversial results, indicating that the physiological role of CAV1 varies according to the cancer type. CAV1 promotes hepatocellular carcinoma cell progression and metastasis through the Wnt/β-catenin pathway [23], whereas it suppresses focal adhesion turnover and migration of metastatic cells [24]. We found that CAV1 and p-JNK increased at 24 h and 48 h after cordycepin treatment (Figure 3A). JNK plays a crucial role not only in death receptor-initiated extrinsic but also in mitochondrial intrinsic apoptotic pathways [25]. After phosphorylation, p-JNK (activated JNK) can activate its downstream transcriptional factors, triggering pro-apoptotic target gene Bax expression as well as inducing cell apoptosis [26] Thus, our results suggest that CAV1-mediated p-JNK upregulation induces A549 cell apoptosis after cordycepin treatment. Because Foxo3a has been identified as a downstream effector of JNK in tumor growth inhibition [27], we assessed whether Foxo3a was changed at 24 h and 48 h after cordycepin treatment. Total Foxo3a was not changed, whereas p-Foxo3a, the inactive form of Foxo3a, was decreased at both time points (Figure 3C), indicating that the active form of Foxo3a had increased in the nucleus. Once p-Foxo3a downregulation occurs, Foxo3a nuclear translocation is facilitated [28, 29]. We found that nuclear translocation of Foxo3a occurred together with the downregulation in the expression of p-Foxo3a at 24 h and 48 h after cordycepin treatment (Figure 3C). These findings suggest that the reduction of p-Foxo3a promotes the nuclear translocation of Foxo3a, inducing the expression of the pro-apoptotic proteins Bax and cleaved caspase-3 and leading to A549 cell apoptosis following cordycepin treatment.

These findings are consistent with those of previous studies [30, 31]. The inhibition of JNK by SP600125 significantly suppressed cordycepin-induced Bax expression (Figure 4A), strongly indicating that JNK regulates the activity of Bax, as well as the expression of cleaved caspase-3 in A549 cells. Next, we investigated whether CAV1 was involved in the positive regulation of p-JNK after cordycepin treatment (Figure 5A). We found that CAV1 overexpression increased the expression of p-JNK, whereas siRNA-mediated inhibition of CAV1 decreased p-JNK, indicating that CAV1 indeed mediated JNK phosphorylation.

The role of DUSPs in both cancer proliferation and cancer resistance is not clear [32]. DUSP5 downregulates members of the MAPK superfamily (MAPK/ERK, SAPK/JNK, p38), which are related with cellular growth and differentiation [33]. Knockdown of Cav-1 attenuates JNK activation through enhanced function of the JNK inhibitor DUSP5 [34]. We found that CAV1-silenced A549 cells treated with cordycepin also showed increased DUSP5 and decreased p-JNK levels, while siRNA inhibition of DUSP5 increased p-JNK (Figure 5A). These findings indicated that CAV1 plays a pivotal role as an inhibitor of DUSP5, and that CAV1-mediated p-JNK upregulation through decreased function of the JNK inhibitor DUSP5 induced A549 cell apoptosis after cordycepin treatment.

The mechanism by which JNK regulates Foxo3a in an Akt-dependent manner after cordycepin is not clear. JNK-mediated phosphatase activities may be related with the Foxo3a dephosphorylation [35, 36]. We investigated whether SP600125 affected Foxo3a expression and phosphorylation. We found that JNK inhibition by SP600125 upregulated the expression of p-Foxo3a at 48 h after cordycepin treatment, suppressing Foxo3a nuclear translocation (Figure 4). Apart from Bax, JNK regulates 14-3-3 protein phosphorylation [37]. Serine phosphorylation of 14-3-3 facilitates the mitochondrial translocation of Bax, a process essential to initiating mitochondrial apoptosis [38]. We found that the inhibition of JNK by SP600125 significantly upregulated cordycepin-suppressed 14-3-3 (Figure 5C). These findings suggest that JNK is involved in the 14-3-3 protein phosphorylation. Because Foxo3a is also a downstream effector of Akt [39], we questioned whether Akt and 14-3-3 are involved in the regulation of CAV1/JNK/Foxo3a. We found that p-Akt and 14-3-3 were downregulated and total Akt was not changed at 48 h in the SP600125-treated A549 cells (Figure 5C). This finding suggested that the regulation of Foxo3a phosphorylation and nuclear translocation by the JNK signaling pathway was induced by p-Akt and 14-3-3. The transcription factor Foxo3a can upregulate pro-apoptotic gene Bax expression and facilitate promote caspase-dependent apoptosis [40]. Additionally, the direct effect of JNK on Bax and consequent Bax mitochondrial translocation are essential to initiating mitochondrial apoptosis [41]. We further investigated whether the inhibition of Foxo3a by siRNA could downregulate the expression of Bax and cleaved caspase-3, and decrease A549 apoptosis. We found that the inhibition of Foxo3a by siRNA reduced the expression of Bax (Figure 5C), indicating that it also significantly inhibits A549 cell apoptosis after cordycepin treatment.

Our findings are consistent with a recent report showing that CAV1 upregulation may generate apoptosis in cancer and that the CAV1/JNK/Foxo3a pathway is involved in A549 cell apoptosis after cordycepin treatment. These results indicate that CAV1-mediated JNK regulation of Foxo3a may be dependent of Akt. Thus, CAV1-mediated JNK regulation of Foxo3a should be considered as a potential therapeutic target. Finally, by using xenograft tumors, we were able to show *in vivo* that cordycepin reduced lung cancer tumor growth and increased the levels of CAV1 and p-JNK, whereas it decreased the level of p-Foxo3a (Figure 6). After xenografted HCC827 and PC9 to nude mice, we compared the size of tumor between cordycepin-treated group and the no-treat control group. Similar to the A549, lots of tumors were occurred in the comparison group, but we could not find any tumor in the cordycepin-treated group. (Supplementary Figure 1). These findings suggested that the reduction of p-Foxo3a promotes the nuclear translocation of Foxo3a, inducing the expression of the pro-apoptotic proteins Bax and cleaved caspase-3, leading to A549 cell apoptosis after cordycepin treatment *in vivo*. Taken together, our results demonstrate that cordycepin has the unique capability of positively regulating Foxo3a nuclear translocation by modulating CAV1/JNK signaling, giving it a pro-apoptotic function. As such, the results suggest that cordycepin has potential as a new drug that could suppress the growth of lung cancer cells.

## MATERIALS AND METHODS

### Reagents and chemicals

Dulbecco's modified Eagle's medium (DMEM) and cordycepin (3’-deoxyadenosine, from *Cordyceps militaris*, Cat. C3394) were purchased from Sigma-Aldrich (St. Louis, MO, USA). Fetal bovine serum (FBS), 1% (w/v) penicillin-streptomycin stock solution, and phosphate-buffered saline (PBS) were obtained from Thermo (Paisley, Scotland, UK). An Annexin-V-FLUOS staining kit was from both Roche Diagnostics GmbH (Mannheim, Germany) and Sigma Chemical Co. Whole cell lysis buffer was from Intron (Seoul, Korea), and transfection reagent Hilymax and cell-counting kit-8 (CCK-8) were from Dojindo (Dojindo, Japan). Antibodies against Foxo3a, p-Foxo3a, p-JNK, caspase-3, p-Akt, and β-actin were from Cell Signaling (Beverly, MA, USA). Antibodies against CAV1, Akt, 14-3-3, DUSP, and Bax, and JNK inhibitor SP600125 were from Santa Cruz (Dallas, TX, USA). Antibody against p-JNK for immunohistochemistry was from Dako (Glostrup, Denmark).

### Cell lines and cell viability assay

The human lung adenocarcinoma lines A549, HCC827, and PC9 were obtained from the American Type Culture Collection (Rockville, MD, USA). The cells were grown in DMEM supplemented with 10% (v/v) FBS and 1% (w/v) penicillin-streptomycin at 37°C under 5% CO_2_ in a humidified incubator. Cells (5 × 10^3^/well) were seeded into a 96-well plate. After a 24-h incubation, the cells were treated with 60 μg/mL of cordycepin for 48 h. Cell viability was assayed as reported previously [42]. In brief, at the end of treatment, 10 μL of CCK-8 solution was added to the cell solution and incubated at 37°C for 1 h. Cell viability was determined by measuring the absorbance at 450 nm using a microplate reader (Sunrise, Tecan, Switzerland). The appropriate dose was determined by evaluating the cytotoxicity of cordycepin after 48 h.

### Cell cycle analysis by propidium iodide (PI)/Annexin V staining

To detect the effect of cordycepin on apoptosis, we analyzed the PI-Annexin V staining pattern using the Annexin V-FLUOS staining kit (Roche Diagnostics). Briefly, cells were treated with cordycepin for 48 h and collected. Suspended cells were centrifuged at 2,000 × *g* for 2 min and incubated at room temperature with 0.2 mg/mL Annexin V-FLUOS and 1.4 mg/mL PI and RNase solution for 15 min under dark conditions. Measurements were conducted on an Image Cytometer (NUCLEOCOUNTER® NC-3000™; Chemometec, Copenhagen, Denmark) with an excitation wavelength of 488 nm and a 530/30-nm band-pass filter to detect Annexin V and a 670 nm high-pass filter to detect PI.

### Microarray analysis

For transcript profiling of cordycepin-treated lung cancer cells, human whole-genome microarrays (Agilent Technology, Palo Alto, CA, USA) were used. Total RNA was extracted from vehicle- or 60 μg/mL cordycepin-treated A549 cells, and labeled with Cy3 and Cy5, respectively. Microarray analysis was conducted according to the manufacturer's protocol as described previously [43]. Data normalization and determination of fold changes in gene expression were done using GeneSpringGX 7.3 (Agilent Technologies). The microarray data have been submitted to the Gene Expression Omnibus database (GEO accession number: GSE81727).

### Gene ontology-based network analysis

To study the biological functions of the regulated genes through their interaction network, we conducted a network analysis by using ingenuity pathway analysis (IPA, http://www.ingenuity.com) to examine the biological functions of the differentially regulated genes and proteins according to ontology-related interaction networks, including apoptosis signaling. Network generation was optimized from the obtained expression profiles when possible, and was aimed at producing highly connected networks.

### CAV1 silencing and overexpression

To overexpress CAV1, we used lentivirus carrying RFP-conjugated full-length CAV1 (Lenti H1.4-cav1/RFP; Bioneer Corp., Daejeon, Korea). The nucleotide sequences of the siRNAs used in this study were as follows: for CAV1, 5’-AGA CGA GCU GAG CGA GAA GCA-3’; for DUSP5, 5’-GGC CUU CGA UUA CAU CAA G-3’; and for Foxo3a, 5’-ACU CCG GGU CCA GCU CCA C-3’. Scrambled control siRNA (Silencer Negative Control 5) was provided by Ambion (Waltham, MA, USA). Lentiviral infection was performed according to the manufacturer's method. Briefly, A549 cells (2 × 10^5^ cells/well) were seeded into a 6-well plate and infected with 1 mL of lentivirus for 8 h. The cells were supplied with growth medium containing 10% FBS and were harvested 48 h later. Transfection of siRNA into the A549 cells was performed using Lipofectamine RNAiMAX reagent (Invitrogen, Carlsbad, CA, USA) in accordance with the manufacturer's instructions. Cells were then treated with 60 μg/mL of cordycepin for 48 h.

### Wound healing assay

The wound healing assay was conducted on control siRNA-transfected and siCAV1-transfected cells. Cells were seeded into a 24-well plate. Then, the cell monolayer was scraped with a pipette tip to create a wound. The cells were treated with 60 μg/mL of cordycepin for 48 h. The plates were imaged using the TissueFAXS system (TissueGontics, Vienna, Austria). Wound closure was analyzed (quantification of the “healed” area and migrated cells). Wound closure analysis was done with with the HistoQuest software (TissueGnostics, Vienna, Austria).

### Immunofluorescence microscopy

A549 cells were seeded on a coverslip in a 12-well plate. The cells were incubated with Foxo3a monoclonal purified mouse IgG1 as a primary antibody overnight at 4°C. In addition, the cells were incubated with fluorescein isothiocyanate-anti-mouse antibody. Fixed cells were washed with PBS and stained with 4,6-diamidino-2-phenylindole at room temperature. The cells were again washed twice with PBS and mounted with mounting solution for observation. Images were acquired using an LSM 710 laser-scanning confocal microscope (Carl Zeiss, Jena, Germany) equipped with a C-Apochromat 40×/1.2 water immersion lens (488 nm Ar laser/505–550 nm detection range). Image data were analyzed with the ZEN 2009 Light Edition software (Carl Zeiss).

### Fractionation and protein extraction

A549 cells were incubated with cordycepin for two days and then fractionated into cytosolic and nucleic fractions as described previously [44]. Briefly, cells were lysed, and the lysates were homogenized and centrifuged at 100,000 ×*g* for 30 min. Pellets were resuspended by sonication, incubated for 30 min at 4°C by rocking, and centrifuged at 100,000 ×*g* for 30 min. The protein contents of the cytosolic and nuclear fractions were determined using a bicinchoninic acid (BCA) assay kit (Thermo Scientific, Rockford, IL, USA) and analyzed by western blotting using anti-Foxo3a antibody.

### Western blot analysis

Regulatory proteins of cordycepin-induced apoptosis were examined by western blot analysis as described previously [43]. After 48-h incubation with cordycepin, proteins were extracted and quantified. Equal amounts of the protein samples (30 μg) were separated by 12% polyacrylamide gel electrophoresis. The resolved proteins were transferred onto nitrocellulose membranes, which were stained with Ponceau S and blocked with 5% skimmed milk in T-TBS (0.1% (v/v) Tween-20 in TBS), followed by incubation with the desired primary antibodies against CAV1 (1:1000), JNK (1:200), p-JNK (1:200), Foxo3a (1:500), p-Foxo3a (1:100), caspase-3 (1:500), Bax (1:1000), Akt (1:1000), p-Akt (1:1000), 14-3-3 (1:1000), DUSP5 (1:1000), and b-Actin (1:2000). All antibodies were used under the same reaction conditions. The blots were washed three times for 5 min each time with T-TBS before incubation with horseradish-peroxidase (HRP)-conjugated goat anti-mouse IgG or HRP-conjugated rabbit anti-goat IgG at a 1:2,000 dilution in TBS containing 5% skimmed milk. The membrane was rinsed 3 times with T-TBS for 5 min each and was developed using an enhanced chemiluminescence system (Thermo Scientific, San Jose, CA, USA) on a ChemiDoc MP system (Bio-Rad, Hercules, CA, USA). The expression level was measured by densitometry using the ImageJ software.

### Tumor xenograft experiment

The animal study was approved by the Institutional Animal Care and Use Committee (IACUC) of the Korea Basic Science Institute (KBSI). Tumor xenografts were established by subcutaneous injection of 1 × 10^6^ A549, HCC827, or PC9 cells suspended in DMEM into the hind limbs of 5-week-old nude (nu/nu Balb/c) mice. The tumor-bearing mice were sorted randomly into 2 groups: vehicle PBS (n=6) and 20 mg/kg cordycepin (n=6). The mice were gavaged once daily for 20 days. The animals were housed in a facility approved by the Association for Assessment and Accreditation of Laboratory Animal Care on a 12-h light/12-h dark cycle with food and water ad libitum. The mice were sacrificed 21 days post-injection. Tumors were fixed with 4% paraformaldehyde in PBS. Tumor volume was evaluated by three-dimensional (3D) ultrasonographic measurement (Philips IU22 Ultrasound, KPI Ultrsound Inc., Yorba Linda, CA, USA).

### Immunohistochemistry

Tumors were removed from the mice and each tumor was cut across the dorsoventral diameter, followed by fixation in ice-cold 10% paraformalin overnight. Fixed tumors were embedded in paraffin and sliced. Paraffin-embedded tissue slices were stained using the following primary antibodies: p-JNK (1:500; Dako), JNK (1:500), p-Foxo3a (1:200), Foxo3a (1:500), and CAV1(1:500). Images were acquired using a Zeiss AxioImagerZ1 microscopy system with a charge-coupled device camera and a TissueFAXSTM automated acquisition system (TissueGnostics, Vienna, Austria). Proteins were quantified as percentages of antibody-positive and marker-positive tumors and depicted as scattergrams. Statistical analysis was performed with the HistoQuestTM software (TissueGnostics).

### Statistical analysis

GraphPad Prism software (GraphPad, San Diego, CA, USA) was used for the statistical analyses. Student's *t*-test was used to assess differences between the control and the cordycepin-treated groups.

## SUPPLEMENTARY MATERIALS FIGURES AND TABLES




