# Supplementary material for: Cordycepin induces apoptosis by caveolin-1-mediated JNK regulation of Foxo3a in human lung adenocarcinoma
Source: Oncotarget. 2017 Jan 14;8(7):12211–24. doi: 10.18632/oncotarget.14661 (PMC5355338; doi:10.18632/oncotarget.14661)
Supplement: Supplementary file 1 [file oncotarget-08-12211-s001.pdf]

Cordycepin induces apoptosis by caveolin-1-mediated JNK regulation of Foxo3a in human lung adenocarcinoma

SUPPLEMENTARY FIGURES AND TABLES

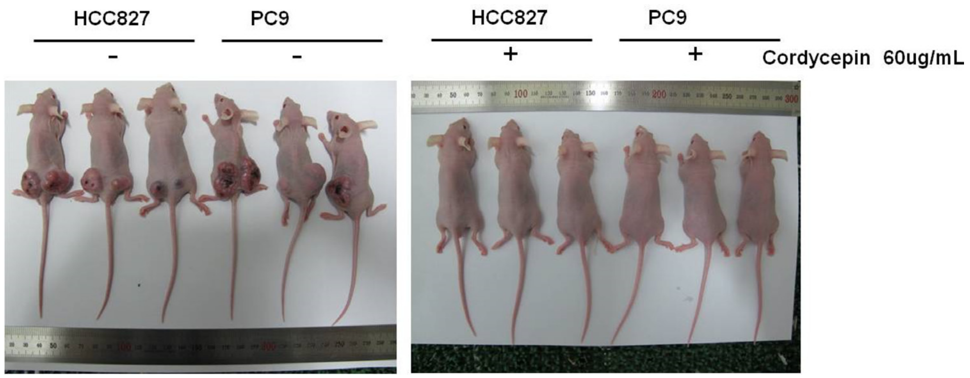

Supplementary Figure 1: Change in tumor appearance in mice after mock therapy with non-reactive components (left) and treatment with cordycepin (right).

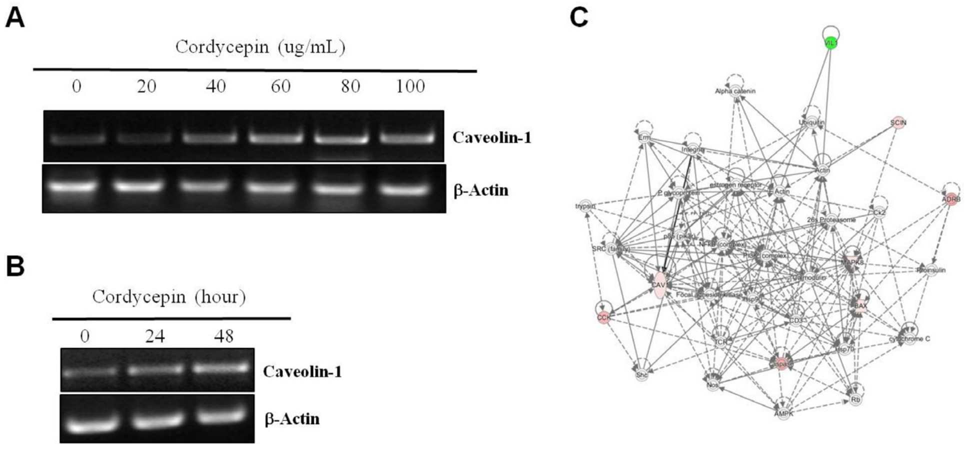

Supplementary Figure 2: Effect of cordycepin on caveolin-1 in A549 cells. Total cellular RNA was analyzed by real-time PCR for caveolin-1. A549 cells in monolayer were incubated with cordycepin in dose **A.** and time **B.** dependent manner. Each value represents the mean ± SEM of three independent experiments. **C.** Signal network of the apoptotic genes in response to cordycepin.

Supplementary Table 1: GeneOntology results on 2-fold up and down regulated genes

See Supplementary File 1
