# Supplementary material for: Cordycepin induces apoptosis by caveolin-1-mediated JNK regulation of Foxo3a in human lung adenocarcinoma
Source: Oncotarget. 2017 Jan 14;8(7):12211–24. doi: 10.18632/oncotarget.14661 (PMC5355338; doi:10.18632/oncotarget.14661)
Supplement: Supplementary file 2 [file oncotarget-08-12211-s002.docx]

Supplementary Table S1

GeneOntology results on 2-fold up and down regulated genes

| Up-regulated genes | |  |  |  |  |  |  |  |  |
| --- | --- | --- | --- | --- | --- | --- | --- | --- | --- |
| Category | Term | Count | % | PValue | Genes | Fold Enrichment | Bonferroni | Benjamini | FDR |
| GOTERM_BP_5 | Signal transduction | 28 | 31.95875 | 0.041317 | 55065, 7130, 219699, 5251, 8875, 22843, 3552, 8651, 6280, 3383, 79627, 6361, 3918, 2069, 2867, 374, 5597, 5138, 4804, 7097, 3569, 6004, 597, 467, 23645, 3604, 80329, 2537 | 1.478154 | 0.955949 | 0.464461 | 35.70182 |
| GOTERM_BP_5 | Apoptosis | 22 | 24.7191 | 0.024527 | 10202, 10116, 3569, 26471, 2537, 6280, 7498, 9518, 1026, 3552, 79094, 8739, 7097, 4804, 3604, 597, 7128, 219699, 23645, 1649, 3383, 3934 | 3.053283 | 0.840804 | 0.458027 | 22.88837 |
| GOTERM_BP_5 | Immunity and defense | 14 | 15.73034 | 0.002866 | 7837, 240, 6289, 285755, 3604, 5005, 3552, 80329, 6280, 3383, 6361, 2537, 3569, 3934 | 2.472659 | 0.191363 | 0.191363 | 2.959612 |
| GOTERM_BP_5 | Cell surface receptor mediated signal transduction | 12 | 13.48315 | 0.092063 | 55065, 79627, 6361, 2069, 219699, 2867, 4804, 8651, 7097, 3552, 3569, 6004 | 1.65856 | 0.999213 | 0.639762 | 63.60994 |
| GOTERM_BP_5 | Cell communication | 10 | 11.23596 | 0.067306 | 6361, 3918, 7130, 5251, 8875, 374, 3552, 3569, 6280, 3383 | 1.91533 | 0.994236 | 0.576566 | 51.77505 |
| GOTERM_BP_5 | Oncogenesis | 6 | 6.741573 | 0.038622 | 79627, 2069, 8148, 1026, 80329, 3934 | 3.153286 | 0.945779 | 0.51745 | 33.78462 |
| GOTERM_BP_5 | Cytokine and chemokine mediated signaling pathway | 5 | 5.617978 | 0.021568 | 6361, 8651, 7097, 3552, 3569 | 4.623918 | 0.800805 | 0.553688 | 20.40448 |
| GOTERM_BP_5 | Cytokine/chemokine mediated immunity | 3 | 3.370787 | 0.095339 | 6361, 3552, 80329 | 5.670384 | 0.999398 | 0.604183 | 64.96105 |

| Down-regulated genes | |  |  |  |  |  |  |  |  |
| --- | --- | --- | --- | --- | --- | --- | --- | --- | --- |
| Category | Term | Count | % | PValue | Genes | Fold Enrichment | Bonferroni | Benjamini | FDR |
| GOTERM_BP_5 | Glycoprotein biosynthetic process | 7 | 6.234655 | 0.008697 | 9514, 9215, 7104, 7903, 84620, 5046, 350 | 6.077927 | 0.950451 | 0.777403 | 11.23319 |
| GOTERM_BP_5 | Apoptosis | 5 | 4.761905 | 0.00416 | 23017, 7429, 10451, 350, 10628 | 7.502441 | 0.761615 | 0.761615 | 5.5275 |
| GOTERM_BP_5 | Protein amino acid glycosylation | 5 | 4.761905 | 0.00416 | 9514, 9215, 7104, 7903, 84620 | 7.502441 | 0.761615 | 0.761615 | 5.5275 |
| GOTERM_BP_5 | Negative regulation of cell differentiation | 4 | 3.809524 | 0.098921 | 4854, 8313, 3400, 8365 | 3.556713 | 1 | 0.988655 | 75.85128 |
| GOTERM_BP_5 | Inorganic anion transport | 3 | 2.857143 | 0.082211 | 115019, 56172, 9022 | 6.195565 | 1 | 0.985241 | 68.97237 |
